# Flagellate Erythema Induced by a Low Cumulative Dose of Bleomycin: A Case Report

**DOI:** 10.7759/cureus.115436

**Published:** 2026-08-30

**Authors:** Kiley B Kirby, Alison Lam

**Affiliations:** 1 Department of Family Medicine, Naval Hospital Camp Pendleton, Oceanside, USA; 2 Department of Dermatology, Naval Medical Center San Diego, San Diego, USA

**Keywords:** bleomycin rash, bleomycin side effect, flagellate erythema, flagellate pigmentation, non-seminomatous germ cell tumor

## Abstract

Flagellate erythema is a rare, distinctive dermatitis that may be the result of treatment with bleomycin or ingestion of shiitake mushrooms. It has also been associated with conditions such as systemic-onset juvenile idiopathic arthritis (Still disease) or dermatomyositis. We present a case of bleomycin-induced flagellate erythema that occurred at a cumulative dosing of 30 mg, which is less than what typically precipitates onset. Bleomycin-associated flagellate erythema is thought to occur due to the low levels of bleomycin hydrolase (BMH) in the skin. This condition is typically self-limiting, with the mainstays of treatment focusing on control of associated pruritus. In this case, improvement was observed at the two-month follow-up, with resolution of pruritus and characteristic progression to hyperpigmentation, which showed areas of fading at four months.

## Introduction

Flagellate erythema is a characteristic dermatitis that can occur in up to 20% of patients following treatment with bleomycin [1]. Bleomycin is a chemotherapeutic drug derived from *Streptomyces verticillus* used alone or in conjunction with other agents to treat lymphomas, some types of squamous cell carcinomas, and testicular cancer [2,3]. Approved by the FDA in 1973, the medication acts on the G2 phase of the cell cycle, specifically inducing the breakdown of single- and double-stranded DNA through the formation of reactive oxygen species [4,5]. The side effects of bleomycin most commonly occur in the lungs and skin, which is thought to be due to their respective decreased levels of bleomycin hydrolase (BMH) [4,6]. The typical presentation of flagellate erythema is dose-dependent, usually occurring after a cumulative dose of 100-300 mg and within 9 weeks of treatment [1].

Flagellate erythema has also been seen in connective tissue diseases (CTD), like dermatomyositis, and CTD mimics such as systemic-onset juvenile idiopathic arthritis (Still disease) [7]. Following the ingestion of mushrooms, most commonly shiitake mushrooms, it is known as shiitake flagellate dermatitis.

While not required for diagnosis, the histopathology of bleomycin-induced flagellate erythema has increased epidermal melanin without an increase in the number of melanocytes [1]. Features commonly seen in both drug-induced and shiitake flagellate dermatitis include spongiosis, dermal lymphocytic infiltrate, and commonly increased eosinophils; features like those found in morbilliform drug reactions [1,7]. However, unlike drug eruptions, the histopathology of bleomycin-induced flagellate erythema does not typically feature necrotic keratocytes [8].

## Case presentation

A 21-year-old, active-duty naval sailor presented to the emergency department (ED) following 1 week of diffuse pruritic rashes. He had undergone radical orchiectomy six weeks prior for the removal of a favorable-risk, nonmetastatic Stage IIC mixed nonseminomatous germ cell tumor (NSGCT). The patient had no additional medical concerns or dermatological history and no known drug allergies. He was prescribed ibuprofen and acetaminophen as needed for postoperative pain. Standard chemotherapy regimens for this type of carcinoma include three cycles of bleomycin, etoposide, and cisplatin (BEP), as initially selected for this patient, or four cycles of etoposide and cisplatin (EP) alone [9]. There was no report of smoking history, and baseline pulmonary function tests and audiogram obtained prior to chemotherapy initiation were within the expected age-adjusted ranges. On BEP Cycle 1 Day 5, 4 days following initial bleomycin exposure, the patient began experiencing generalized pruritus and was given 25 mg of diphenhydramine by mouth (PO); he tolerated the remainder of the treatment without issue. However, later that day, he began to develop the first signs of dermatitis (Figure 1). At this time, 4 days following initial bleomycin exposure, the patient had received a cumulative dose of 30 mg of bleomycin, 1,150 mg of etoposide, and 230 mg of cisplatin.

**Figure 1:**
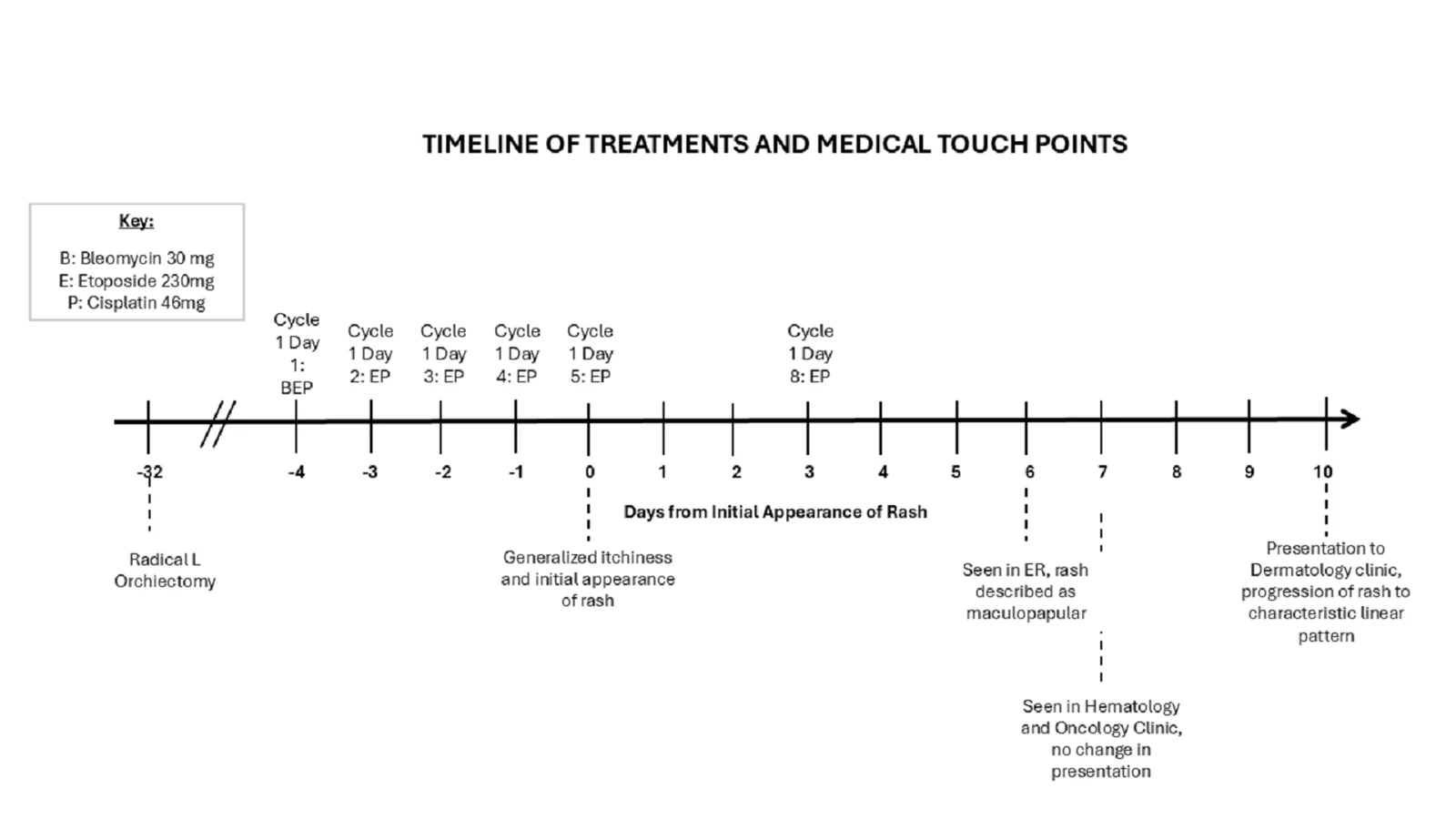
Chronological account of medication administration and follow-up in relation to rash presentation

Six days later, he presented to the ED concerned for continued pruritus and skin changes; he was afebrile, well-appearing, and hemodynamically stable. Laboratory findings included leukopenia without elevations in erythrocyte sedimentation rate or C-reactive protein, mildly elevated liver enzymes, and intact renal function. Physical exam consisted of a pustular rash on the back and maculopapular rashes on the chest, buttocks, posterior hands, bilateral elbows, and bilateral shins. Due to his ongoing chemotherapy treatments, there was concern for disseminated varicella zoster virus, drug eruption, as well as bleomycin-induced flagellate erythema. There was no concern for acute bacterial infection given his lack of fever or elevated inflammatory markers. The patient was given clobetasol 0.05% ointment twice a day, valacyclovir 1 g three times a day for seven days, and cetirizine 10 mg daily, as needed.

He followed up in the Hematology and Oncology clinic the next day, where he was continued on the same regimen and referred to the Dermatology clinic. The patient presented to the Dermatology clinic on day 10 following the initial presentation of the rash. Herpes simplex virus testing returned negative; however, varicella zoster virus (VZV) PCR was not available for review, as the sample was not collected at initial presentation in the ED. While the initial physical description of the dermatitis from the ED may have favored morbilliform drug reaction, no photos were available for review. The patient had no use of ibuprofen or acetaminophen in the week preceding, but his chemotherapy regimen could have caused a hypersensitivity reaction. However, by the time the patient was evaluated in the Dermatology clinic, it had progressed to scattered linear erythematous plaques on the neck (Figure 2) and extremities (Figure 3), consistent with bleomycin-induced flagellate erythema. Other systemic diseases that may have flagellate erythema as a cutaneous manifestation were considered. However, the clinical picture pointed to bleomycin exposure due to the patient’s lack of additional systemic symptoms, such as fevers and arthritis associated with Still’s disease or proximal muscle weakness, and more common findings, such as heliotrope signs and Gottron’s papules, as seen in dermatomyositis. Biopsy samples were not obtained to avoid an unnecessary procedure; the diagnosis was made clinically in the context of his ongoing chemotherapy treatment and characteristic physical exam.

**Figure 2:**
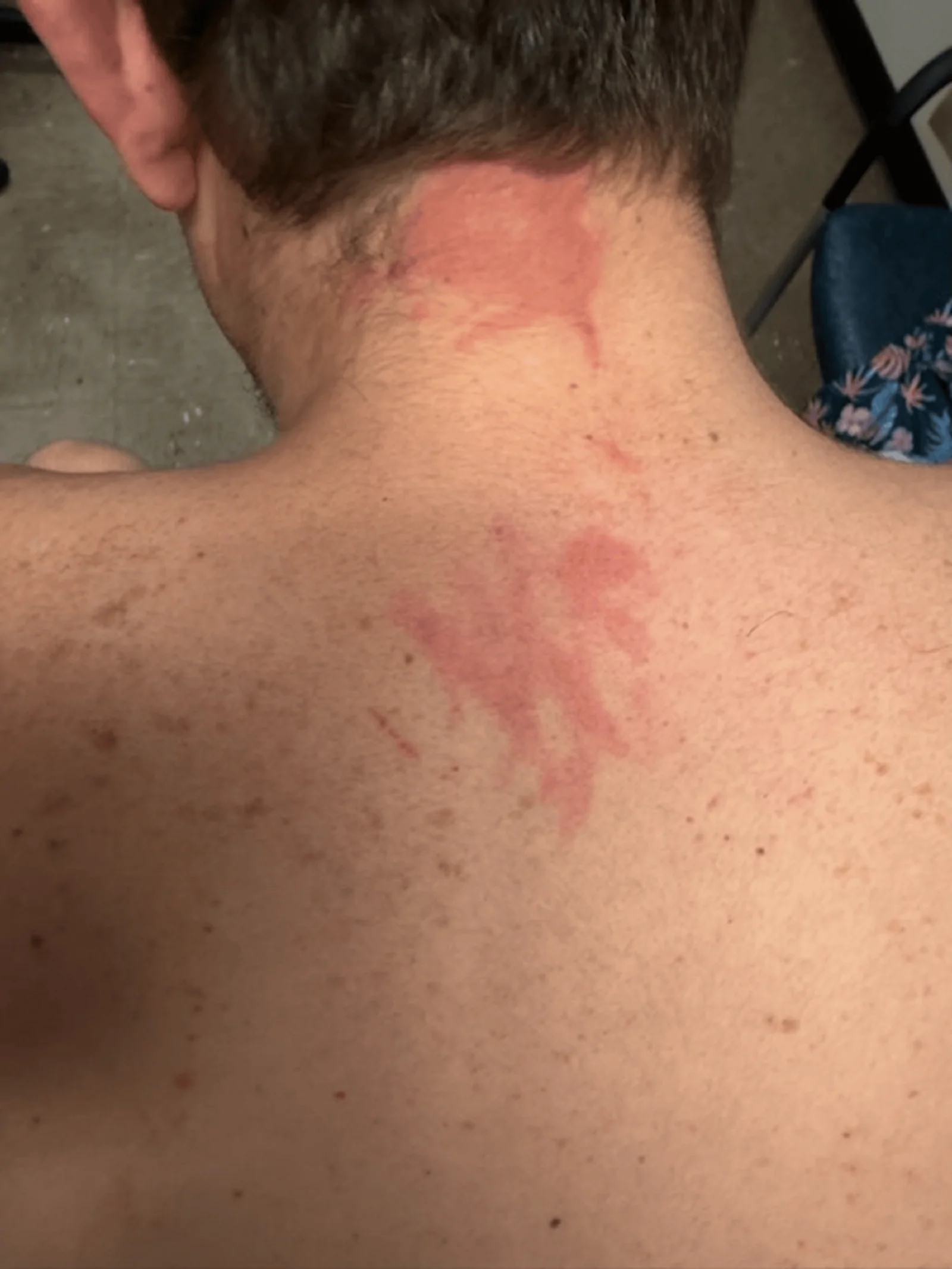
Excoriated, flagellated, brightly erythematous patches on the posterior neck consistent with bleomycin-induced flagellate erythema

**Figure 3:**
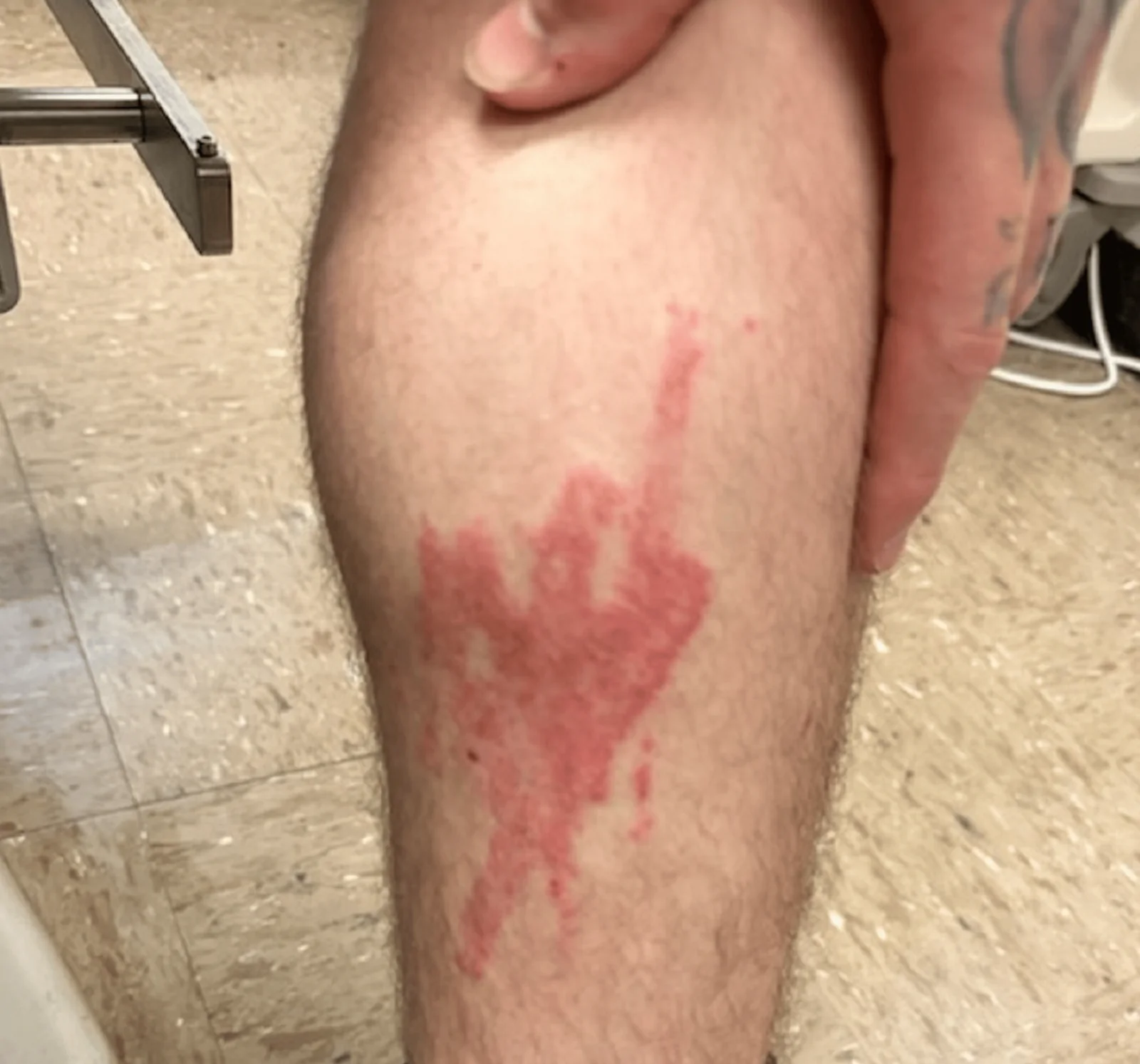
Excoriated, flagellated, brightly erythematous patches on the posterior calf consistent with bleomycin-induced flagellate erythema

He was continued on clobetasol and cetirizine as well as valacyclovir due to an inability to definitively rule out disease with partial treatment, although the physical exam did not favor a VZV infection. His regimen was augmented with desonide 0.05% cream in case of facial involvement and nightly diphenhydramine, as needed. Due to the patient’s sensitivity to bleomycin, his oncology team transitioned to EP alone for four courses, including the initial cycle with bleomycin. Follow-up in the Dermatology clinic the next week revealed progression from salmon-colored patches to dusky pink; his pruritus was inadequately controlled, and so he was transitioned to tacrolimus for both the face and extremities. At the two-month follow-up, the lesions were asymptomatic and characteristically hyperpigmented (Figure 4). Resolution of hyperpigmentation typically occurs three to four months following discontinuation of bleomycin [1]. At four months, this patient had no new rashes, and some areas of the hyperpigmentation had faded.

**Figure 4:**
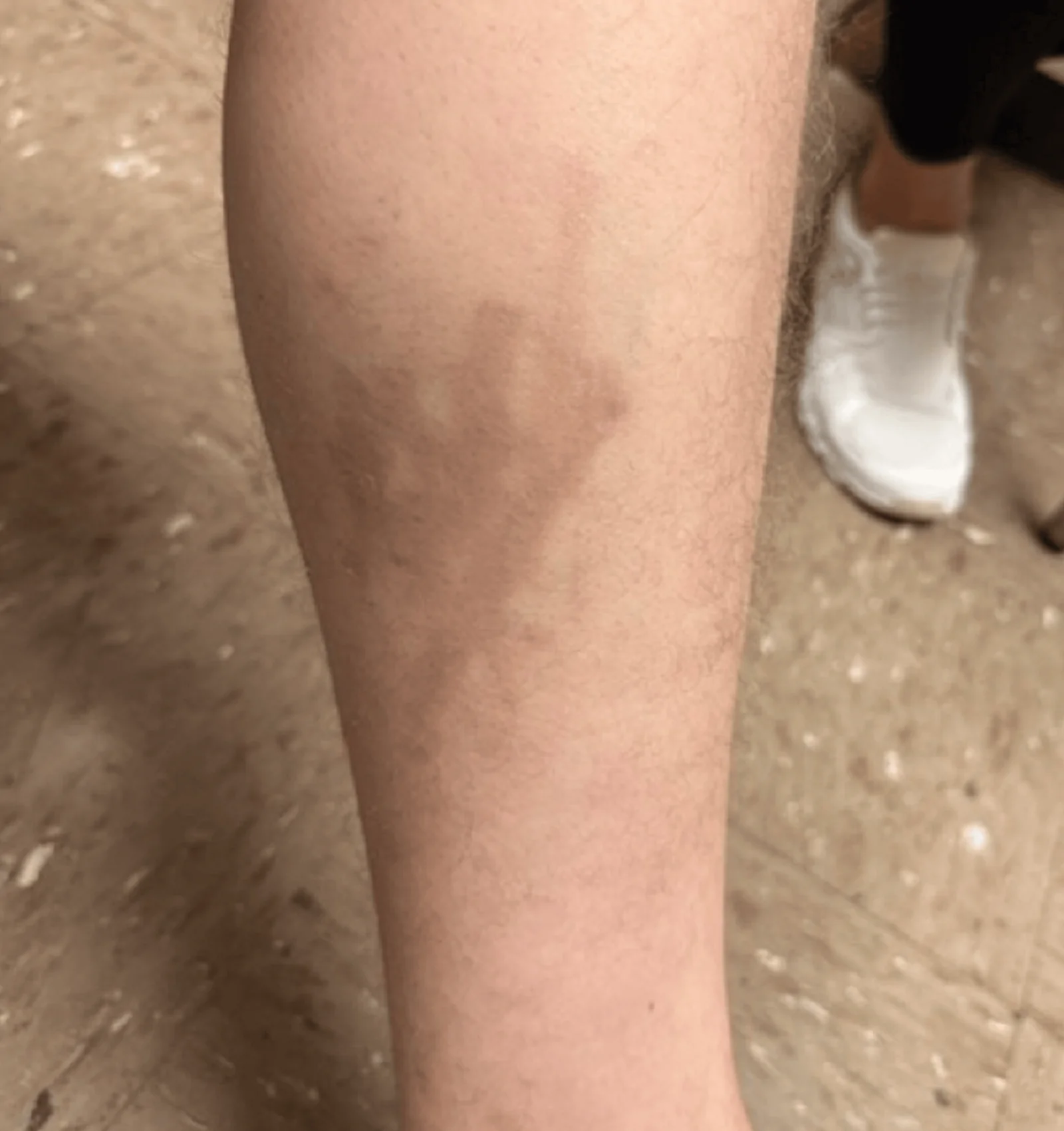
Hyperpigmented flagellated patches on the posterior calf as seen at the two-month follow-up

## Discussion

This case demonstrates flagellate erythema occurring in a patient with a total cumulative dose of only 30 mg of bleomycin, which is below the characteristic threshold of 100-300 mg [1]. While atypical, additional cases have been seen at similar doses of 15-30 mg [1,10,11]. The pathogenesis of flagellate erythema has not been fully defined, but it may be due to an increase in melanogenesis, changes in pigment secondary to increased inflammation, or even BMH polymorphisms, which have been seen could increase susceptibility for the early appearance of flagellate erythema [11-13], Additionally, the patterns and typical locations of lesions suggest the process may be precipitated by scratching; however, many patients have typical dermatological findings without pruritus, and the dermatosis has not been reproduced by scratching [14].

While there is no established link for bleomycin-induced flagellate erythema as a predictor for lung toxicity, such as bleomycin-induced pneumonitis (BIP) in humans, there is a theoretical relation due to the decreased levels of BMH in both the skin and lungs [4,6]. Early studies involving mice bred with a lack of BMH showed an increased incidence of spontaneous dermatitis and increased sensitivity to treatment with high levels of pulmonary fibrosis [15]. Flagellate erythema occurs at a higher incidence than BIP, 10-20% compared to 10% of patients treated with bleomycin, including various patient populations and cancer types [16]. For BIP, the FDA identifies increased risk at a cumulative dosing of greater than 400 mg, while no direct dose-response relationship has been established between pulmonary and cutaneous toxicity; flagellate erythema typically occurs at lower treatment levels.

If there is early presentation of bleomycin-induced flagellate erythema, some clinicians may choose to adjust treatment regimens based on potentially increased risk of additional sequelae. However, this remains a clinical decision and the presence of cutaneous toxicity may not require discontinuation of therapy. There are no specific treatments for bleomycin-induced flagellate erythema apart from controlling pruritus, and pigmentation typically fades three to four months following completion of therapy [1].

## Conclusions

Bleomycin-induced flagellate erythema is a distinct, linear, hyperpigmented cutaneous eruption that can occur in up to a fifth of patients treated with bleomycin. This patient presented with flagellate erythema following a cumulative dosing of bleomycin less than typically reported in the literature. The presence of this cutaneous finding at a lower dose may suggest an increased susceptibility to adverse effects; however, no direct causal relationship has been established, and this requires further investigation. Early clinical recognition of bleomycin-induced flagellate erythema can prompt adjustments to chemotherapy regimens to allow for subsequent improvement of the dermatitis. However, the presence of cutaneous findings alone does not require treatment change and should only be used as part of multidisciplinary clinical decision-making.
